# Hospital managers’ experiences of conducting a root cause analysis: a case study following a sentinel event

**DOI:** 10.3389/frhs.2025.1566335

**Published:** 2025-04-29

**Authors:** Silje Liepelt, Ralf Kirchhoff

**Affiliations:** Department of Health Sciences, Faculty of Medicine and Health Sciences, Norwegian University of Science and Technology, Ålesund, Norway

**Keywords:** patient safety, qualitative research, root cause analysis (RCA) method, sentinel event, guideline adherence, Norway

## Abstract

**Background:**

Root cause analysis (RCA) is a method used in healthcare to systematically identify and address underlying causes of adverse or sentinel events to enhance patient safety and mitigate risks. This study explores hospital managers' experiences of conducting an RCA process following a sentinel event in which a baby unexpectedly died during labor at a Norwegian hospital in 2021.

**Method:**

The study employed a qualitative, exploratory single-case design, which involved conducting nine semi-structured interviews and analyzing documents such as the Norwegian RCA guideline, the final RCA report, and internal procedures and standards. The interviews were conducted between May and August 2021. Thematic analysis was used to organize and interpret the transcribed data. The research addressed the following question: *What were the hospital managers*’ *experiences with conducting a root cause analysis?*

**Results:**

Two main themes emerged. The first theme*, challenges of and strategies for ensuring compliance with the Norwegian RCA Method,* captures the wide range of challenges managers experience, ranging from practical application to communication breakdowns, role ambiguity, and meeting regulatory compliance. The second theme, *emotional burden and support*, underscores the emotional strain managers endured as they navigated the grief of the personnel involved, communicating with the bereaved family, and collaborated with external agencies during the investigation.

**Conclusion:**

The findings highlight the need for more precise role definitions, better resources, and stronger emotional support systems to strengthen RCA processes. Although national RCA guidelines provide a valuable framework, real-world constraints and unique circumstances often require adaptive approaches. This study emphasizes managers’ pivotal role in bridging the gap between regulatory expectations and organizational realities, underscoring the need for both practical and emotional support to ensure effective RCA implementation in sentinel events.

## 1 Introduction

Patient harm from unsafe care is recognized as a significant global health concern and is one of the leading causes of death and disability worldwide (1). International studies indicate that despite considerable efforts, quality and safety in healthcare remain substantial challenges, with high rates of adverse events among hospitalized patients (2–5).

Investigating sentinel events is essential to patient safety and quality improvement in healthcare (6–8). In this paper, we address the role of healthcare managers in hospitals by focusing on their experiences in conducting a root cause analysis (RCA) in Norway. Healthcare managers have a moral and legal responsibility to ensure high-quality patient care and continuously seek improvements. Positioned to influence policy, systems, and procedures while shaping the organizational and social context, healthcare managers are crucial in maintaining and enhancing care standards. Consequently, it is widely recognized that healthcare managers are pivotal in ensuring quality care and patient safety, making it one of their top priorities (9).

Research on managers' experiences with conducting an RCA is gradually expanding, providing valuable insights into the topic. Existing studies on this topic can be broadly categorized into five main groups:

1) *Retrospective studies:* This category includes studies in which managers, often referred to interchangeably as “managers” and “leaders,” were interviewed or surveyed about their past experiences with RCA. Studies in this group include work by Abreu, Freysteinson, Clutter, & Aulbach (10), Bowie, Skinner, & de Wet (11), ledema, Jorm, & Braithwaite (12), and Kok, de Kam, Leistikow, Grit, & Bal (13). These studies highlight challenges such as navigating regulatory expectations, balancing team support with objectivity, and facing resource limitations when retrospectively implementing RCA to analyze sentinel events.
2) *Real-time observations and interviews:* This category encompasses studies in which managers were observed and interviewed while actively conducting an RCA, providing insights into their behaviors and decision-making processes during the analysis. The research by Nicolini, Waring, and Mengis (14) falls into this group, revealing how real-time observations capture the strategic and practical elements of RCA management that might be missed in retrospective studies.
3) *Perceptions of non-managers:* Some studies focus on healthcare staff who do not hold managerial roles but are directly impacted by RCA processes. These studies, including work by Liepelt, Sundal, & Kirchhoff (15), Seys, Scott, Wu, & Gerves (16), and Willis et al. (6), highlight staff perceptions of managerial roles in RCA. Findings from these studies suggest that non-managerial staff view adequate managerial support as essential to the RCA's success, particularly for fostering a non-punitive culture that encourages open communication and shares learning from adverse events.
4) *Literature reviews on healthcare professionals:* Literature reviews, such as that by Seys et al. (16), examine the broader context of healthcare management in RCA, highlighting the critical role of managers in mitigating the emotional and psychological impact on staff involved in sentinel events. These reviews emphasize that managers can foster a supportive environment that shifts the focus from blame to systemic learning, thereby enhancing the overall effectiveness of RCA.
5) *Reviews on technical performance in RCA*: The literature also covers technical performance in RCA. Reviews like the one discussed in Patient Safety & Quality Healthcare (17) indicate that RCA outcomes often depend on managerial involvement, tool selection, and implementation practices. These reviews suggest that for RCA to achieve optimal outcomes, managers must ensure adequate training, consistent methodology, and alignment with broader healthcare goals.

Some studies, such as Abreu et al. (10), identify challenges related to knowledge, education and training gaps. Additionally, the authors emphasize enabling nurses to contribute meaningfully to the analysis. Other studies stress the need for managers, especially those responsible for assembling an RCA team, to recognize and understand the inherent knowledge disparities and asymmetry among different roles within the health personnel and the collection and examination of corroborating evidence (13, 14). The latter implies evaluating the composition of the RCA team and the methods by which participants are encouraged to share their experiences. Kok, de Kam, Leistikow, Grit, & Bal (13) suggest that managers consider arranging for healthcare professionals to be interviewed by their colleagues in the same field, as this peer-to-peer interaction could yield more insightful and candid feedback. A literature review also indicates the need for supportive managers after an adverse event (16).

Braithwaite et al. (18) identify challenges like time constraints, lack of resources, uncooperative colleagues, and differences between professionals' approaches that can complicate RCA. These challenges are highly relevant for managers for several reasons: (a) Managers are responsible for ensuring that RCAs are completed efficiently. If time is limited, it can impede the implementation of RCA, leading to less effective problem-solving and decision-making processes. (b) Managers control the use of resources within organizations. Implementing RCA requires diverting healthcare personnel's time from other tasks, which can be a constraint, especially given that national guidelines recommend adequate resourcing for RCA. A lack of resources can thus hinder the process. (c) When health personnel involved in a sentinel event are unwilling to participate in the RCA process, it creates a barrier to understanding the factors that contributed to the incident, both for the individuals and the system. This research suggests that managers should motivate and support employees to promote their involvement in the investigation. (d) Differences in professional backgrounds can lead to varying perspectives and approaches to problem-solving, which can be valuable in conducting an RCA. While this variety can be beneficial, it can present a challenge if not managed effectively. This research suggests that managers should facilitate purposeful communication and collaboration among team members with different professional backgrounds during the RCA process and consider how to communicate the RCA results.

Although there is some literature on managers' experiences with RCA following sentinel events, studies remain limited and largely reflect contexts rather different from Norway, including the USA (10), the UK (14), Scotland (11), and Australia (12). Few studies have employed case design to explore managers' experiences with RCA after the sentinel event in which they participated. This study aims to connect these experiences to the recommendations outlined in the Norwegian RCA guideline, which emphasizes a systematic approach to understanding and preventing adverse events, fostering a safety culture, and promoting continuous quality improvements in healthcare (19).

The following sections begin with an outline of crucial aspects of RCA-related sentinel event policies. They are followed by an overview of the Norwegian regulatory context that informs our case study. We then present the methodology and conduct a discussion that integrates findings with previous research.

## 2 Study context

### 2.1 Sentinel event policy and manager roles associated with RCA

The Joint Commission, a U.S.-based nonprofit organization, accredits healthcare organizations to improve healthcare quality and patient safety (20). In 1996, The Joint Commission adopted the Sentinel Event Policy to support hospitals in investigating and analyzing severe patient safety incidents. Sentinel events are defined as patient-safety events that result in death, permanent harm, or severe, temporary harm (8). These sentinel events serve as warning signals of potential systemic issues that could lead to future harm if unaddressed (21). The policy supports healthcare organizations in preventing future harm through in-depth systemic evaluations.

The complexity of sentinel events is often difficult to reveal due to several compelling factors related to care processes involving many stakeholders across different health system levels. Additionally, hospitals vary in how they define, investigate, and report sentinel events (22). To create an effective safety culture, Øyri et al. (23) emphasize the need for more substantial management commitment and recognition of quality and safety as integral to the operational culture of healthcare organizations.

### 2.2 Root cause analysis: A framework for investigating sentinel events

RCA broadly refers to a range of methodologies and tools designed for the retrospective and structured investigation of adverse events, near misses, and sentinel events (24, 25). RCA aims to improve patient safety by reducing the number of medical errors and adverse events. Originating from high-reliability industries, it has become the mandated methodology for investigating adverse events in most health systems (6, 26). A typical RCA investigation tries to identify root causes and contributory factors that are thought to have either directly or significantly contributed to the adverse event (26, 27).

RCA provides a framework for hospitals to identify system and process vulnerabilities that could contribute to risk. Since its introduction in the early 20th century, various approaches have been developed to improve the RCA process and formulate effective solutions. However, a study by Kellogg et al. (28) found that most proposed solutions were weak actions unlikely to prevent future mistakes. Although RCA is recognized as essential for patient safety (29, 30), there is still a significant knowledge gap regarding its practical implementation in healthcare settings (31).

### 2.3 Challenges in implementing effective RCA solutions

Achieving effective quality improvement throughout RCA requires robust management support and consistent application. The active involvement of managers is pivotal, as they oversee the RCA process from initiation to completion (see Table 1). Their responsibilities include assembling a multidisciplinary team, providing necessary resources, and ensuring a thorough investigation. In Norway, RCA generally follows an eight-step process outlined by the guideline (19)

**Table 1:** The eight standard chronological steps in the Norwegian RCA process with assigned responsibilities in this case.

RCA step	Tasks	Person responsible
Step 1: Initiate analysis	Define the scope and objectives	Process owner, with support from Department Heads and Section Managers
Step 2: Collect facts	Gather relevant data and details of the incident	Analysis leader and analysis team, with input from Department Heads and Section Managers
Step 3: Describe the sequence of events	Map the sequence leading to the incident	
Step 4: Identify underlying causes	Analyze data to find root causes	
Step 5: Propose actions and follow-up methods	Develop action plans and methods to monitor their implementation	
Step 6: Write the final report	Document findings and recommended actions	
Step 7: Decide on actions	Approve actions for implementation	Process owner, with input from Department Heads and Section Managers
Step 8: Evaluate and follow up on actions	Monitor the implementation of actions and assess their effectiveness	Process owner, Department Heads and Section Managers

The process owner is responsible for initiating the RCA, clearly defining the scope and objectives, and ensuring follow-up actions are implemented effectively. The analysis leader guides the investigation team through data collection, mapping out the sequence of events, identifying underlying causes, and recommending actionable improvements (19). This methodical approach aligns with the *Regulation on Management and Quality Improvement requirements in Health and Care Services,* emphasizing the necessity of systematic quality management to ensure patient and user safety (32).

### 2.4 Establishing and leading the RCA team

The initial step of the RCA process involves establishing an interprofessional team to define and investigate the incident (15, 33). This team typically includes healthcare professionals from various disciplines, such as physicians, nurses, administrators, and quality improvement experts (20). The analysis leader plays a pivotal role by ensuring the team adheres to the structured RCA methodology. This includes coordinating meetings, facilitating communication across departments, and guiding the team through steps 2–6 of the RCA (15, 19).

Once the RCA team has completed these steps, the process owner is responsible for steps 7 and 8. Step 8 typically involves implementing and monitoring the corrective actions identified during the analysis and is closely linked to quality improvement. Step 8 in the RCA process applies quality improvement principles to address identified issues and sustain improvements systematically over time.

A tool or method like the RCA guideline is designed to demonstrate reasonable reliability and validity. Validity refers to how well a technique measures what it claims to measure (34), while a reliable technique would produce consistent results regardless of who applies it. Despite the theoretical framework of RCA, considerable variability exists in its practical application within healthcare (26). This variability encompasses team composition, meeting frequency, RCA duration, adherence to RCA steps, incident severity, and prioritizing RCA findings for action. In addition to the process owner, other managers are involved in the RCA process, even if they do not have formal roles as process owners. These managers provide valuable insights and expertise from their respective fields, which are crucial for identifying root causes and developing effective actions. Their participation ensures a holistic approach and the anchoring of actions across the organization.

### 2.5 The Norwegian regulatory framework

Norway has no formal “sentinel event policy” like the U.S. Joint Commission (8). However, the country has established healthcare policies and regulations to ensure patient safety and manage adverse events. These policies are supported by national guidelines aimed at improving healthcare quality and safety (19). Over the past decades, several initiatives have been introduced to strengthen hospitals' focus on patient safety (23), including the *National Action Plan for Quality and Patient Safety (2019–2023)* (2) and the “Leading the way to zero” initiative, which seek to eliminate patient harm by implementing reliable healthcare processes (2). These initiatives stress the importance of investigating healthcare failures to prevent future incidents.

Norwegian hospitals are required to provide employees with relevant training in quality improvement methodologies (2, 35). The national reporting system, introduced by parliamentary decision in the early 1990s, marked a milestone in building a formal healthcare oversight framework (36). In 2010, this system was expanded into a more comprehensive framework for reporting adverse events, with the goal of strengthening learning and improving patient safety.

In 2016, the Norwegian Directorate of Health published RCA guidelines inspired by Bagian's work at the U.S. Department of Veterans Affairs (VA) (19, 37). These guidelines, adapted from a publication by the Swedish Association of Local Authorities and Regions (38), provide methodological support to improve patient safety through RCA. In the Norwegian context, the RCA process involves eight stages (see Table 1), with clearly defined roles and responsibilities that align with regulatory and organizational expectations. The Norwegian Directorate of Health also offers RCA training courses, which have been widely adopted in the Norwegian healthcare system (19).

Oversight of RCA implementation is provided by authorities such as the Norwegian Board of Health Supervision and the Offices of the County Governors. In sentinel events, timely internal reporting and notification to the Norwegian Board of Health Supervision are critical. According to Section 3–3a of the Specialist Health Service Act (39), specialist health services must report serious and unexpected adverse events. This section, introduced in 2010, responds to incidents within specialist health services where patients have suffered significant harm or death under circumstances in which these outcomes would not have been expected or foreseen.

In 2017, Norway introduced a new regulatory framework to support local quality and safety initiatives within healthcare services (32). This framework, *Regulation on Management and Quality Improvement in Health and Care Services,* expands management's responsibilities in several key areas:

1) Overall accountability: Management at the top level is responsible for ensuring that the organization's activities are planned, executed, evaluated, and corrected following the regulation.
2) Systematic quality management: Managers must establish a quality management system that systematically controls organizational activities and supports continuous improvement and patient safety.
3) Employee involvement: The framework emphasizes the importance of management's role in employee involvement. Management must ensure employees participate in the management system and quality improvement work, which includes providing necessary training and competence development.
4) Tailored documentation: Management must ensure that all activities and measures are documented in a manner adapted to the organization's size, nature, and risk conditions. This regulation applies to managers in specialized hospital settings and primary healthcare services.

Based on these initial insights about what is known about managers' experiences in conducting an RCA and their formal responsibilities within the Norwegian RCA legal framework and guidelines, we raise the following research questions: *What were hospital managers' experiences with conducting a root cause analysis (RCA)?*

## 3 Materials and methods

### 3.1 Data sources

This study analyzed multiple data sources to understand the RCA process comprehensively, including the final RCA report, national and internal RCA guidelines, and in-depth interviews with key hospital managers. The final RCA report detailed the incident's circumstances, contextualizing the RCA process within incident-specific outcomes and challenges. National RCA guidelines and hospital-level procedures also provided insights into the structural and procedural frameworks guiding RCA implementation. In-depth interviews with managers added nuanced perspectives on decision-making, coordination, and adaptations within the RCA process.

### 3.2 Study design

Building on a previous study exploring RCA teams' experiences (15), this study focuses on hospital managers' experiences with RCA. Unlike RCA team members, managers hold distinct roles and responsibilities within the RCA process, providing a unique perspective. We employed a qualitative, exploratory single-case design (40) to examine managers' roles and perspectives regarding the RCA process in a Norwegian hospital. This design allowed for an in-depth exploration of the RCA process following a sentinel event, covering the preparation, execution, and follow-up stages. Data collection included in-depth interviews with nine hospital managers responsible for conducting RCA, supplemented by document analysis of the Norwegian RCA guidelines, the hospital's internal RCA report, and relevant internal procedures. This combination of data sources provided a comprehensive understanding of the RCA process and its context.

### 3.3 Setting and sample

The study was conducted at a medium-sized Norwegian hospital—the first to respond positively after being contacted during preparation to carry out an RCA following a sentinel event. With approximately 6,000 employees, the hospital has conducted RCA since 2016, performing it 2–4 times yearly with varying team composition for each analysis. One hospital agreed to participate, allowing the research team to interview their staff after a fatal sentinel event occurred. During the RCA process, we identified nine managers as key informants. These managers supported second victims involved in the sentinel event during the RCA process (see Table 2). The informants were crucial participants within the healthcare organization, holding management roles in various departments directly involved in the sentinel event. Their selection was based on their direct involvement in the RCA processes and their ability to provide insight into how RCA is conducted as perceived within their clinical settings.

**Table 2:** Characteristics of study participants.

Profession	Position in Healthcare Organisation	Background	Experience with Root Cause Analysis
Physician	Clinic manager	The process owner is responsible for ensuring the RCA's effectiveness and meaningful impact on patient safety. The RCA is initiated, corrective actions are approved, and implementation is monitored (Steps 1, 7, and 8 in Table 1). This role entails strategic oversight and accountability, guiding the RCA from start to finish and ensuring actions align with hospital safety goals.	Experience from two full-scale RCA processes and two mini-RCAs. No formal training in the methodology.
Physician	Head of Paediatric Department	The section manager for the physician in the department is responsible for personnel and follow-up with the staff. Medical advisor for the department head (who is a nurse), as well as former Head of the Quality Council. Worked in the Paediatric Department for 20 years. Chief physician for 12 years. Works 60% clinically and 40% administratively and is a former Internal Patient and Quality Safety Committee member.	No formal training in RCA, but had received internal training from the quality advisor. Had been interviewed in an earlier RCA.
Midwife	One of two section managers of the Maternity and Gynaecological Outpatient Clinic, with primary responsibility for the Gynaecological Outpatient Clinic	Personnel responsibility for about 40 employees. Has worked as a manager since 2008, including as an assistant section leader.	Trained in the RCA methodology through the Directorate of Health in 2019. Had not used the methods before this event.
Nurse	Head of Department for the Emergency, Intensive Care Unit and Surgery Department	Oversees seven sections: Emergency Department, Intensive Care Unit, Operating Room Nurses, Intensive Care Nurses, Anaesthesiologists, Sterilisation Unit, and Day Surgery. Responsible for about 300 employees across various sections. Formal leadership education. Master's degree in health administration.	Had participated in approximately 6–7 RCAs. No formal training in the methodology, but had attended a 3-hour workshop at the Norwegian Patient Safety Conference.
Obstetrician and Gynecologist	Section manager for physicians in the Department of Obstetrics and Gynaecology	Head of Department Physician in the Maternity Ward. Specialization in Obstetrics and Gynaecology. 40% administrative tasks and 60% clinical work. Thirty years of experience in the healthcare organization.	Trained in the methodology through the Directorate of Health in 2019. Had conducted no previous RCAs.
Midwife	One of two section managers of the Maternity and Gynaecological Outpatient Clinic, with primary responsibility for the Maternity ward	Trained as a midwife in 2014. Worked for about one year as a section manager.	No formal RCA training. Had never conducted an RCA before.
Nurse	Section manager of the Neonatal Intensive Care Unit	Responsible for about 30–50 employees. Shares responsibility with an assistant section leader, who acts as deputy if the section leader is absent. Worked at the department as a nurse for about ten years. Further trained in leadership.	Had worked for about one year as a section leader. No experience with RCA. No internal training or formal training in the methodology.
Public health nurse	Head of Department for Paediatric Medicine and Rehabilitation	Oversees four hospitals’ cross-sectional pediatric departments, wards, outpatient clinics, and postpartum visits. Further trained in economics and leadership. Previously worked as a section manager.	Had participated in two RCAs before. Limited involvement. No formal training in the methodology.
Anaesthesiologist	Section manager for the Anaesthesiologists and the pain clinic	Completed specialist training in anaesthesiology in 2014. Senior Consultant in Anaesthesiology. Shares responsibility with an assistant section leader. Shifts in air ambulance service. 50% on-call duty and nominally 40% administrative position.	Had participated in two mini-RCAs in the clinic. No formal training in the methodology. No internal training.

We used purposive sampling, including nine hospital managers who were vital in supporting staff involved in the sentinel event. These informants, each responsible for managing those affected or engaged in the RCA process, provided insights into the organizational context and RCA process (see Table 2).

### 3.4 Data collection

Data collection took place from May to August 2021. The first author initiated the process by contacting Norwegian hospitals to identify RCA teams utilizing the RCA methodology in line with national guidelines. Based on the responses, four hospitals were selected to receive an invitation to participate. Each hospital received an email containing a study description, an invitation to participate, a consent form, and the authors' contact information. Only one hospital—coincidentally preparing to carry out an RCA during the recruitment period—agreed to participate.

Due to the COVID-19 restrictions on social contact, all interviews were conducted digitally via Teams. The first author conducted all interviews, which lasted approximately 1.5–2 h. All interviews were audio-recorded with participants' consent and transcribed verbatim to ensure accuracy. A semi-structured interview guide directed the discussions, covering themes such as leadership and culture, systematic improvement and organizational competence, systems and structures for improvement, RCA participation and competence, employee involvement, and the implementation of action plans. This interview guide was explicitly developed for this study, and neither the guide nor findings from this research have been previously published. The guide is available as a Supplementary Appendix 1.

### 3.5 Data analysis

We employed thematic analysis to identify, analyze, and report themes within the dataset (41). The process involved six key steps: (1) familiarization with the data, (2) generating initial codes, (3) searching for themes, (4) reviewing themes, (5) defining and naming themes, and (6) writing the report.

Initially, we familiarized ourselves with the data by thoroughly reading each interview, which provided an overall sense of the material and allowed us to note preliminary ideas and patterns. We then examined each data item systematically, identifying information relevant to our research question and assigning code labels (see Table 3). As relevant text segments emerged, we determined whether to apply an existing code or create a new one. These codes evolved throughout the analysis, enabling us to capture various meanings within the data.

**Table 3:** Thematic coding and pattern identification—example.

Transcript excerpt	Code	Overacting theme
“Unfortunately, we can't do anything more here; we must step back.’ It's a difficult decision, but I've always received good support from colleagues and others when making such decisions. Rarely do you stand alone in making these decisions. In any case, I haven't, and neither did he. We usually consult with colleagues to ensure that the decision feels supported before making such difficult choices.” “It's always tough to follow up with parents who have lost a baby, of course! But it's comforting to know you've done everything possible to achieve the best possible outcome. And then there's the responsibility of saying, ‘Unfortunately, we can't do anything more. We have to step back now.”	Teamwork and colleague support Supporting families during sentinel events	Emotional burden and support

As our analysis continued, we listened to the interviews, read through transcribed text, and refined our codes, allowing our understanding to deepen. We developed codes to identify common patterns—even when specific text segments conveyed different meanings and applied latent codes to uncover underlying patterns beyond the explicit content. NVivo 14.23.2 (46) software supported basic text-handling tasks throughout this process (42).

In the next phase, we combined related codes to explore their relationships and identified overarching themes that connected different parts of the data. In our final analysis, we systematically reviewed these themes to ensure they accurately represented the observed patterns and provided a comprehensive understanding of the dataset. This rigorous approach underscored the importance of thoroughly engaging with the data, capturing the diversity of interpretations and the complexity within the material.

### 3.6 Ethical considerations

The study adhered to the ethical standards outlined in the World Medical Association's Declaration of Helsinki (43). The Norwegian Centre for Research Data (NSD), now known as SIKT, ensured that the research project complied with Norwegian privacy legislation (project number 562024) following the requirements of the Act concerning Personal Data and the General Data Protection Regulation (GDPR). Participant confidentiality and anonymity were maintained throughout the research process. All data collected were securely stored with access control on secure servers provided by the Norwegian University of Science and Technology (NTNU). The interviews were anonymized during the transcription to protect the participants' privacy and ensure strict adherence to ethical standards.

Participants provided voluntary consent before the interviews and received detailed information about the study's purpose and procedures, and their rights, including the right to withdraw at any time without consequence.

## 4 Results

The results provide an overview of the themes related to hospital managers' experiences with conducting the RCA, directly addressing the research question through two main themes and their associated sub-themes. First, we briefly describe the incident that led to the RCA being conducted, followed by the themes identified through thematic analysis of the RCA process following the incident.

### 4.1 The incident

Our analysis of the RCA team's final report reveals a high-risk birth case involving a woman with gestational diabetes and a prior cesarean section who was admitted for labor induction. She initially received an epidural with limited effect, necessitating a second epidural, after which she began showing symptoms of a high spinal block, including muscle paralysis, respiratory distress, and hypoxia. An emergency cesarean was performed immediately, and while the baby was delivered quickly, it was pale and unresponsive. Resuscitation briefly restored the baby's heartbeat, but signs of severe oxygen deprivation were evident. Despite intensive care efforts, the baby's condition remained critical, and treatment was withdrawn the following day, with the infant being declared deceased approximately 12 h after birth.

### 4.2 Theme: challenges and strategies in ensuring compliance with the Norwegian RCA method

The study's central theme, *challenges and strategies in ensuring compliance with the Norwegian RCA Method*, delves into the complex experience of hospital managers in adhering to national guidelines throughout the RCA process. The findings highlight varying levels of familiarity with RCA guidelines among managers, reliance on quality advisors to bridge knowledge gaps, and practical obstacles that make compliance difficult. Managers demonstrated awareness of their regulatory responsibilities, yet their commitment to meeting these standards was compromised by resource constraints and time pressures– revealing a disconnect between policy expectations and real-world capabilities.

#### 4.2.1 Awareness and understanding

Managers exhibited varying levels of familiarity with the Norwegian RCA guidelines. While some had a basic understanding, others relied heavily on the quality advisor for detailed guidance and application. The quality advisor served as an essential resource, bridging knowledge gaps and helping managers adhere to guidelines. One manager mentioned, “Of course, I consult the guideline when there are things one is unsure about or curious about, but I must say that we have relied heavily on our quality advisor since she has training in this area. I do have the guidelines, but my familiarity with them is limited. To be honest, I probably rely a lot on the quality advisor when I have questions.”

Managers generally expressed a commitment to aligning with national healthcare policies and acknowledged RCA as a vital tool for patient safety. However, the degree of engagement often depended on the frequency of incidents and available resources.

The varying levels of managers' awareness and understanding of the RCA guidelines and the role of quality advisors address a knowledge gap. The reliance on quality advisors highlights managers' limited awareness and understanding of the guidelines. A contradiction here is that managers are expected to be familiar with the guidelines, yet they heavily depend on quality advisors, indicating a discrepancy between expected and actual knowledge levels.

#### 4.2.2 Practical application and challenges

The practical implementation of RCA varied, with managers facing challenges such as time constraints and resource limitations. Nevertheless, RCA's importance in improving patient safety was widely acknowledged. One manager noted, “We were fortunate to have the methodology introduced at the department and clinic levels. Our quality advisor thoroughly reviewed the methodology with us, and we tried to extend its use by presenting it in departmental meetings and with section leaders for broader dissemination. While I have reviewed the handbook myself, I believe that the in-depth guidance provided by our quality advisor and the professional department has been exceptionally valuable.”

Formal training and hands-on experience with RCA were inconsistent across managers. Those with more training and experience found RCA implementation easier, while others struggled with competing priorities. As one manager acknowledged, although she had not consulted the guidelines for several years, reviewing them could be beneficial if she needed to conduct a short version of the RCA without support from the quality department. Another manager remarked, “I have read the guidelines for the national RCA course, but I can't say that I remember much of it.”

Despite differences in experience, training, and familiarity, managers acknowledged the value of RCA as a systematic tool for investigating sentinel events and enhancing patient safety. They appreciated its emphasis on identifying system failures rather than assigning individual blame. This sub-theme illuminates the difficulties in implementing RCA, which are influenced by varying levels of training and experience. Managers with more training found RCA easier to apply, while those without training struggled with practical challenges. Although there is an expectation that formal training should enhance implementation, practical challenges remain, indicating that training alone may not be sufficient.

#### 4.2.3 Navigating process ownership and collaborative dependencies

The process owner's role in initiating and overseeing the RCA process is central to regulatory compliance. However, findings suggest a complex reality in fulfilling this accountability, where the process owner must navigate a web of dependencies and organizational constraints to meet RCA requirements effectively. While formally responsible for guiding the RCA from initiation to final action steps, the process owner often relies on quality advisors and other managers for essential procedural and methodological support, underscoring the difficulty of maintaining independent oversight.

This collaborative reliance points to a structural gap: the process owner's role, though defined as authoritative, is practically interdependent, complicating their ability to ensure compliance autonomously. Several managers noted that heavy dependence on quality advisors for regulatory guidance highlights an imbalance, as the process owners find themselves stretched between formal RCA obligations and the practical need for external expertise. For instance, the process owner noted the challenge of reinforcing reporting standards among clinicians, emphasizing that persuading physicians to report incidents to authorities often involves complex negotiations.

The interdependence between the process owner and other managers also impacts clarity and efficiency in RCA execution. Some informants described overlapping roles that led to delays and miscommunications, especially when interpreting reporting criteria. These dynamics underscore a more significant issue: without more precise role delineation and support mechanisms, the process owner's capacity to enforce compliance is frequently compromised by the need for broader team collaboration.

#### 4.2.4 Role ambiguity, communication breakdown, and regulatory compliance

Managers reported confusion during the RCA process due to unclear roles and responsibilities, often leading to communication breakdowns, delays, and inefficiencies. Managers depended on quality advisors' support and highlighted their reliance on guidance. This dependence underscored the need for broader role clarification and training across the organization.

While managers were aware of regulatory requirements for RCA, they struggled to meet these standards due to time constraints, staffing shortages, and the complexity of sentinel events. This gap between regulatory expectations and practical realities was a recurring challenge.

Staff—particularly physicians—were often hesitant to reporting incidents to external authorities. Concerns about blame, legal repercussions, and the perception of criminal implications contributed to this reluctance, revealing a general discomfort with external scrutiny. The process owner shared, “I have to argue with the doctors about why they should report to the police.”

Several managers also described how police involvement in the RCA process halted progress and complicates a demanding situation. One manager described confusion and a lack of procedural clarity around reporting incidents to the police, with delays in notifications due to unclear responsibility. Another reflected on the heightened stress for staff and management, who faced concerns over accountability and intense scrutiny during police investigations.

Internal disagreements emerged between managers and staff regarding whether and when external regulatory authorities should be notified. Some healthcare personnel, especially doctors, felt that too many incidents were being reported, while the process owner believed that reporting was insufficient. This discrepancy created frustration and confusion, particularly regarding the lack of clarity on when to involve the police. Healthcare professionals differ in their interpretations of the incident reporting criteria and the procedures for notifying authorities. Some professionals adopt a narrow view, believing reporting is only necessary if criminal activity is suspected. This interpretation, particularly among doctors, led to tension between medical staff and managers. Managers emphasized that reporting should adhere to established criteria, regardless of individual suspicions of wrongdoing.

This sub-theme highlights the tension between the intent to comply with regulations and the practical obstacles that make full compliance challenging. Managers often find it impossible to achieve regulatory compliance due to practical limitations, revealing a gap between policy expectations and real-world execution. Resistance to external reporting—motivated by staff fears of blame and potential legal consequences—can create a conflict for managers between internal safety objectives and external accountability requirements.

### 4.3 Theme: emotional burden and support

The second theme focuses on the emotional burden that hospital managers experience during sentinel events. It highlights the importance of teamwork, debriefing, and support systems to help them navigate the challenges such events may lead to.

#### 4.3.1 Teamwork and colleague support

This sub-theme highlights the critical role of teamwork and colleague support in managing the emotional burden that hospital managers face during sentinel events. Managers commonly consulted colleagues to confirm difficult decisions, relying on this collaborative approach to ease the emotional strain. As one manager explained, “Unfortunately, we can't do anything more here; we must step back. It's a difficult decision, but I've always received good support from colleagues and others when making such decisions. Rarely do you stand alone in making these decisions.” Managers emphasized that they seldom face difficult decisions alone during the RCA process. A supportive team, including nurses and other professionals, is perceived as crucial for making informed decisions and providing emotional support. This collaborative approach helps mitigate the emotional weight of these decisions, ensuring that managers feel supported and less isolated.

#### 4.3.2 Emotional toll on staff and support from managers and the occupational health service

This sub-theme addresses the emotional toll on staff of sentinel events and subsequent police questioning, highlighting the crucial role of managerial support during these periods. Managers recognize the significant stress that police investigations impose on staff and emphasize the need for comprehensive support systems. One manager remarked, “We’ve had employees go through police questioning. It's stressful, and we need to support them better.” “People feel like they’re being scrutinized even when they’ve done their best.” Ensuring staff are accompanied during police interrogation and providing ongoing emotional support were seen as critical aspects of care for healthcare workers.

Additionally, managers identified a need for improved psychological support services for staff involved in traumatic incidents. “There has been much criticism that the healthcare facility does not have its team ready to handle reactions from employees.” Current resources, such as the occupational health service (BHT), were viewed as insufficient, and managers suggested implementing more structured support mechanisms, like crisis teams, to help staff cope with the psychological aftermath of sentinel events. However, they also recognized that financial constraints could limit the feasibility of establishing these services. Despite this limitation, managers considered such a support system vital for protecting the long-term mental health of staff and addressing the impacts of traumatic incidents.

#### 4.3.3 Supporting families during sentinel events

Norwegian guidelines emphasize the importance of involving patients and families in the analysis of sentinel events. This involvement is essential for identifying all contributing factors and addressing the affected individuals' emotional and psychological needs.

Managers reflected on the complex challenges of supporting parents in tragic situations, particularly when facing difficult ethical decisions. These dilemmas often involve decisions about whether to continue treatment, especially when survival could mean severe brain damage for the child. As one manager shared, “It's always tough to follow up with parents who have lost a baby, of course! But it's comforting to know you've done everything possible to achieve the best outcome. And then there's the responsibility of saying, ‘Unfortunately, we can’t do anything more, we have to step back now.’”

These ethically challenging decisions were made collaboratively, with managers relying on the support of colleagues. During interviews, managers emphasized the delicate balance between doing everything possible to save a child and accepting the ethical responsibility of ending treatment when it is in the best interest of the child's quality of life. They also highlighted the importance of consulting with others to ensure these decisions were carefully considered and ethically sound.

## 5 Discussion

Our study describes and analyzes the experiences of hospital managers in Norway in conducting root cause analysis (RCA) after a sentinel event. The first theme, *challenges and strategies in ensuring compliance with the Norwegian RCA method*, underscores managers’ difficulties in adhering to national RCA guidelines. Although the interviewed managers were committed to patient safety, they frequently faced practical limitations—such as time, staffing, and resource shortages—that hindered compliance. They described navigating confusion and resource constraints, exposing a tension between standardized guidelines and the realities of practice. This discrepancy suggests that national RCA guidelines alone may be insufficient to achieve optimal compliance without adequate organizational support.

A notable finding concerns the process owner's role (see Table 1). Although this role is central to RCA accountability, process owners often rely heavily on quality advisors and other managers for methodological and procedural guidance. This reliance indicates an inherent interdependence in the RCA process, where the process owner's ability to ensure organizational dynamics and available expertise moderate compliance. Our findings emphasize the need for broader role clarity and enhanced support for process owners, as ambiguities in roles and dependencies may compromise the consistency and effectiveness of the RCA. These findings are consistent with prior research indicating that RCA execution may be hindered when roles lack clear delineation (10).

Our findings also raise questions about the adequacy of the Norwegian RCA guidelines. Although designed and adjusted to fit Norway's healthcare system, our results indicate that the Norwegian guidelines may not sufficiently address the complexities of decision-making under the real-world constraints managers must handle. This raises a fundamental question: Does the current framework adequately define and support the manager's role in RCA, or is there a need for more explicit guidance on how managers should navigate the complexities of the RCA process? The managers we interviewed found the guidelines lacking in practical instructions for handling role conflicts and interdependencies. Managers and process owners often grapple with ambiguities regarding their responsibilities and how best to fulfill their roles. Also, Kellogg et al. (28) have found that many RCA guidelines need to be revised, emphasizing the limitations of current approaches. The latter finding underscores the need to strengthen RCA methods in Norway to make them more applicable to managers. Here, we will assume that this may also be relevant for countries with health systems different from the Norwegian one. Further, we suggest that process owners receive comprehensive training and adequate resources to develop these methods.

Another finding from this study shows that resistance to external reporting, especially from physicians, reflects a broader discomfort with accountability mechanisms that are perceived as punitive. Managers noted the difficulty of balancing internal safety objectives with the regulatory pressures for transparency, suggesting a need for policies that support a non-punitive reporting culture. This tension between internal goals and external accountability echoes findings from previous studies highlighting the importance of fostering a safety culture that values learning over blame (9).

In line with other studies (18), the managers we interviewed experienced a lack of resources for conducting the RCA. Lack of resources, which affected staff availability and ability to meet the need for ongoing training, was perceived as an obstacle to achieving compliance with the RCA guidelines. Also, Parand et al. (9) revealed that many hospitals struggle with resource constraints and competing priorities, which hinder their ability to consistently follow quality and safety guidelines. We note that managers who participated in the RCA wish to act fully engaged but recognize that they must make compromises and adjustments. The ambitious goal of regulatory compliance seems out of reach. This illustrates the difference between the ideal standard “state-of-the-art facility” delivering the highest possible standards in healthcare and the reality of what can be achieved given these limitations.

Moreover, our findings suggest that the Norwegian emphasis on a collaborative, non-punitive approach—while commendable—adds a layer of complexity for management. Managers must balance being supportive, team-oriented leaders while ensuring regulatory compliance and process efficiency. This dual expectation can be difficult to manage, especially when the guidelines do not provide clear strategies for handling role conflicts or interdependencies. Thus, there is an evident need to reconsider whether the Norwegian RCA guidelines should offer more nuanced descriptions and practical strategies for how managers should execute their roles effectively under real-world constraints.

The second theme, *emotional burden and support*, underscores the emotional dimension experienced by the managers in the study, as they had to manage the grief of the involved personnel, communicate with the bereaved family, and cooperate with external agencies investigating the event. The managers we interviewed experienced that they both gave and received emotional support. They were not alone in bearing the strain of standing in such demanding processes or anchoring decisions following this sentinel event. However, they raised criticism of the organization's ability to support its employees by offering psychological help through the hospital's occupational health service. The hospital was not prepared with a strategy for dealing with the acute need for mental support after the sentinel event.

The *emotional burden and support* theme underscores the human dimension of RCA processes. Managers oversee the technical aspects of an RCA and play a vital role in supporting staff through the emotional strain of sentinel events. The managers we interviewed experienced the need for psychological support and debriefing as critical, mainly when external investigations, such as police inquiries, increase the stress on staff. This finding aligns with earlier ones, suggesting that emotional and psychological support is vital in maintaining staff morale and ensuring continued participation in quality improvement processes (16). The study also shows the importance of teamwork and peer support, as managers rarely make decisions alone but instead rely on collaborative processes to navigate the challenges of sentinel events.

## 6 Conclusion

Our findings suggest a need for streamlined processes and improved resource allocation to ensure the effectiveness of RCA procedures and other safety protocols. By addressing these practical constraints, healthcare organizations can achieve their ideal standards of quality care. Notably, our results underscore the importance of clear role definitions, particularly for process owners, and the establishment of more robust support systems to bridge the gap between compliance goals and the operational realities of RCA implementation.

Overall, the findings reflect the complexity of conducting RCAs in real-world settings and emphasize an active role managers play in navigating both formal accountability requirements and the emotional burden that sentinel events place on staff. Managers were not only responsible for ensuring regulatory compliance, but also for acting as emotional anchors and decision-makers in uncertain and high-pressure contexts. This dual demand reveals how a managerial agency is experienced at the intersection of procedural expectations and human impact.

To better align RCA practices with organizational capacity, future initiatives should ensure adequate resources, clear role delineation, and structured psychological support for both managers and staff. Further research should explore how RCA practices can be embedded more sustainably within healthcare organizations and examine their long-term impact on patient safety outcomes. Including frontline clinical staff in future studies would offer valuable insight into the practical challenges of RCA and help shape policies that are both realistic and inclusive of all stakeholder perspectives. In particular, attention should be given to strategies that bridge the gap between policy and practice, including the development of structured systems for psychological support during and after sentinel events.

## Data Availability

The raw data supporting the conclusions of this article are available from the corresponding author upon reasonable request and in line with ethical and legal requirements.

## Supplementary material

Supplementary Material Appendix 1 Semi-structured interview guide.

Description of Data This document contains the semi-structured interview guide used in the study titled “*Hospital managers’ experiences conducting a root cause analysis: A case study following a sentinel event.”* The guide includes open-ended questions to explore hospital managers’ experiences and reflections on the root cause analysis process, patient safety culture, quality improvement work, and organizational learning following a sentinel event.
