# Supplementary material for: Hospital managers’ experiences of conducting a root cause analysis: a case study following a sentinel event
Source: Front Health Serv. 2025 Apr 29;5:1566335. doi: 10.3389/frhs.2025.1566335 (PMC12069439; doi:10.3389/frhs.2025.1566335)
Supplement: Description of Data — This document contains the semi-structured interview guide used in the study titled “Hospital managers’ experiences conducting a root cause analysis: A case study following a sentinel event.” The guide includes open-ended questions to explore hospital managers’ experiences and reflections on the root cause analysis process, patient safety culture, quality improvement work, and organizational learning following a sentinel event. [file Datasheet1.pdf]

Interview guide for managers

Leadership and culture

1. How was information about the incident collected?

    a) What criteria determine whether a case with serious consequences leads to an RCA in this healthcare organization?

    b) How was this specific RCA initiated?

    c) How was the preparation for the analysis leader conducted?

    d) How were the analysis team and analysis leader selected?

2. Describe how you, as a manager, influence patient safety and quality improvement in the clinic:

    a) How would you describe the patient safety culture in the organization?

    b) How do you work with the work environment in the clinic?

3. Has the pandemic affected the execution of incident analysis?

Systematic improvement work and organizational competence

1. All hospitals are legally required to have a quality committee. How is this organized in your organization?
    - a) Is it divided into different levels?
    - b) How does the committee get information about serious adverse events?
2. How do you evaluate which incidents should be analyzed using the RCA methodology?
3. How does your clinic work with quality improvements?

    a) Which model do you use?

    b) What other methods, besides incident analysis, are used for learning after adverse events in the organization?

Systems and structures (infrastructure for improvement work)

1. What systems/routines does the organization have for disseminating and learning from the RCA results across organizational boundaries?
2. What is the significance of method selection regarding economy and resource utilization?
3. How was the task communicated within the organization's leadership group, quality department, patient safety team, or similar?

    a) Were the findings presented in meetings or other networks?

b) Were the results shared outside the relevant units, and if so, which publication channels were used?

c) Were other units involved in this work?

4. What experiences do you have with the Norwegian guidelines and methodology described in the handbook?
5. What are your experiences using the national guidelines as a handbook for conducting RCA?
6. What are your experiences with RCA as a methodology for learning after sentinel events?

Competence and participation in root cause analysis

1. How were employees followed up after an RCA was initiated?
2. How and to what extent were those directly involved in the sentinel event included in the incident analysis?
3. I am interested in knowing more about how patients, relatives, and employees affected by sentinel events are followed up by the hospital:

    a) How is the involvement of patients and relatives carried out?

    b) How do employees' direct patient-related experiences influence your leadership?

    c) What considerations were made for those involved in the sentinel event?

4. Was debriefing initiated after the incident?

    a) Who conducted this, and how was it carried out?

Implementation and change competence

1. How did employees in the clinic react when the RCA was initiated?
2. Explain how you/your team determined which measures to implement after completing the RCA:

    a) What experiences do you have with implementing proposed measures in the action plan?

    b) Did you encounter resistance/barriers to implementation?

    c) Were all measures from the RCA attempted to be implemented?

    d) Were the measures implemented on a small scale before being broadly introduced within the organization?

    e) How was the implementation carried out?

f) Could the implementation have been done differently?

3. What are your experiences with changes being adopted or rejected in the clinic?
4. What are your expectations of employees regarding change/implementation?
5. How were the measures evaluated and followed up?

    a) What are your experiences with recognizing that a change is an improvement?

    b) What systems/routines does the clinic have for testing implemented changes?

6. Were risk areas identified after the evaluation was completed?

    a) How were they followed up, if applicable?

7. Describe the advantages/disadvantages of the methodology for organizational learning after serious adverse events in the clinic.
